# Neuroprotective potential of Afzelin: A novel approach for alleviating catalepsy and modulating Bcl-2 expression in Parkinson's disease therapy

**DOI:** 10.1016/j.jsps.2023.101928

**Published:** 2023-12-18

**Authors:** Khalid M. Alharthy, Summya Rashid, Hasan S. Yusufoglu, Saleh I. Alqasoumi, Majid Ahmad Ganaie, Aftab Alam

**Affiliations:** a Department of Pharmacology and Toxicology, College of Pharmacy, Prince Sattam Bin Abdulaziz University, Al-Kharj 11942, Saudi Arabia; b Department of Pharmacognosy and Pharmaceutical Chemistry, College of Dentistry and Pharmacy, Buraydah Private Colleges, Buraydah, Al-Qassim 51418, Saudi Arabia; c Department of Pharmacognosy, College of Pharmacy, King Saud University, Riyadh 11451, Saudi Arabia; d Department of Pharmacology & Toxicology, College of Dentistry and Pharmacy, Buraydah Colleges, 51418 Buraydah, Saudi Arabia; e Department of Pharmacognosy, College of Pharmacy, Prince Sattam Bin Abdulaziz University, Alkharj 11942, Saudi Arabia

**Keywords:** Parkinson's disease, Dopaminergic neurons, Bcl-2, Reserpine, Antioxidant

## Abstract

The lost dopaminergic neurons in the brain prevent mobility in Parkinson's disease (PD). It is impossible to stop the disease's progress by means of symptoms management. Research focuses on oxidative stress, mitochondrial dysfunction, and neuronal degeneration. Exploration of potential neuroprotective drugs against prosurvival B-cell lymphoma 2 (Bcl-2) protein is ongoing. An investigable cause behind PD, as well as preventive measures, could be discovered considering the association between such behavioural manifestations (cataleptic behaviours) and PD. The compound Afzelin, known to guard the nervous system, was chosen for this study. The study was done on rats divided into six different groups. First, there was a control group. The other group was treated with Reserpine (RES) (1 mg/kg). The third group received RES (1 mg/kg) and levodopa (30 mg/kg). The remaining three groups were given RES (1 mg/kg) in conjunction with Afzelin at the following doses: 5 mg/kg, 10 mg/kg, and 20 mg/kg. Cataleptic behavior and mobility in rats was assessed using the rotarod, open field, and modified forced-swim tests. thiobarbituric acid reactive substances (TBARS), nitric oxide (NO), biogenic amines, and Bcl-2 level in rat tissue homogenates were considered. According to the study's findings, the rats treated through co-administration of RES and Afzelin improved significantly in their cataleptic behaviours and locomotor activity. In addition, administering Afzelin itself caused Bcl-2 expression, which could have some neuroprotection properties. This study provides meaningful information on the effectiveness of Afzelin in handling catalepsy and other degenerative neurologic disorders. As a result, other studies need to be conducted to establish the reasons behind the reactions and determine the long-term effects of Afzelin on these conditions.

## 1 Introduction

Parkinson’s disease (PD) is a chronic neurodegenerative disorder that results mainly in motor impairment. This condition is expressed in a group of specific symptoms, for instance, tremor, rigidity, bradykinesia, reduced postural stability, and impaired motor coordination (Kouli et al., 2018). Hereditary and environmental factors cause Parkinson's (Warner et al., 2003) and symptoms gradually worsen. Parkinson's commonly begins with a hand tremor, muscular stiffness, movement speed concerns, walking and balance challenges, speech changes, facial expression alterations, fatigue, sadness, and sleep irregularities that can accompany this tremor (Magrinelli et al., 2016).

In PD, the substantia nigra loses dopaminergic neurons. Dopamine, a neurotransmitter that aids in coordination, is depleted by this loss of cells. Motor dysfunction, tremor, stiffness, bradykinesia, and balance and coordination are defining symptoms of PD (Su et al., 2023). Current treatment options for PD focus on symptom management rather than stopping or reversing disease progression. PD's motor deficits are caused by this neurodegenerative process, highlighting the need for medicines that restore or preserve dopaminergic neurons and dopamine levels.

From a pathological point of view, oxidative stress, mitochondrial malfunction, and impaired protein handling kill neuronal loss in PD (Malkus et al., 2009). The degradation of dopaminergic neurons in Parkinson's disease is commonly related to the process of apoptosis. Programmed Cell Death in an organised way and involving several entities like B-cell lymphoma (Bcl-2) family, apoptotic peptidase activating factor (Apaf-1), and caspase (Erekat, 2018). The anti-apoptotic protein Bcl-2 and other apoptosis-related elements have been investigated extensively. Nowadays, no doubt that it is the most crucial reason causing such robust safety against cell death is its unambiguous location within the mitochondria (Abdin and Hamouda, 2008). One of the essential proteins is Bcl-2, which blocks the release of cytochrome *c* from the mitochondria, preventing the activation of caspases. The caspase is essential in the intricate apoptosis process, leading to cell death (Tsujimoto, 1998). However, in PD, Bcl-2 levels may be dysregulated, leading to an imbalance in pro-apoptotic and anti-apoptotic factors (Liu et al., 2019, Van der Heide and Smidt, 2013). Bcl-2’s function in PD could shed light on dopaminergic neuron loss and neuroprotective therapies.

Despite the therapies available, patients with PD still experience significant disability. Current treatments aim to manage symptoms, but cannot stop or reverse disease progression. Standard treatment involves levodopa with carbidopa to replenish dopamine levels and alleviate motor symptoms. However, long-term use of levodopa can lead to motor fluctuations and dyskinesias. Increasing the dosage of levodopa increases the risk of side effects such as hallucinations and cognitive impairment. In addition, levodopa mainly affects motor symptoms with minimal influence on non-motor disturbances such as cognitive deficits and mood disorders. Current research explores other therapeutics aimed at sustained and consistent stimulation of dopaminergic activity. One of the most important and relevant fields of research is related to nonmotor symptoms in PD. This helps improve the efficacy of the medication and improves people with Parkinson’s quality of life. Therefore, it means that when looking for therapy alternatives, these drugs do not have neuroprotective properties and may cause undesirable side-effects.

Afzelin has strong therapeutic properties and even interacts with some physiological processes in human cells. Complex pharmacology is involved in this compound with antioxidant, anti-inflammation, anti-carcinogenics, and antimicrobial properties that can be used as the foundation of new drugs. Nature may become an accessible source of treatment for various diseases without drug addiction and its harmful impact. One of them is afzelin, derived from medicinal herbs and shrubs (Akter et al., 2022). Hence, there is need to study more about the clinical effectiveness of afzelin for the management of PD. Therefore, afzelin should provide an outstanding nutritional component in the sector of natural health and exercise sciences. It is an effective antioxidant and anti-inflammatory that has received attention of scientist and researchers. For our precious bran’s sake, hopefully, its pre-clinical studies give a glimpse on its potential protective effects. Afzelin’s journey goes on because it gives the hope of new discoveries which could pave the way for a neuroprotective therapeutics (Devi et al., 2021a, Devi et al., 2021b, Oh et al., 2021). The element attracts a lot of interests since it regulates cell death pathways while promoting neuronal survival that is critical in diseases like PD (Oh et al., 2021).

Using reserpine (RES)-induced catalepsy as an experimental model to understand some aspects of PD. RES reduces dopamine in the brain and subsequently develops catatonic symptoms, which closely mimic the symptoms experienced in Parkinson’s. The validity of this technique is demonstrated as being essential in understanding diverse clinical conditions underlying PD (Cunha et al., 2022, Hubbard and Trugman, 1993, Lima et al., 2021, Patil et al., 2012). The RES-induced catalepsy will help in revealing some neurochemical and neurophysiological alterations related to PD (Li et al., 2023, Singh et al., 2003, Soares et al., 2021). The model is useful in several therapeutic procedures when examining how efficient neuroprotective materials can be found, identifying new targets of materials, and establishing motor symptom and sign management techniques.

This study aims at determining if Afzelin could regulate Bcl-2 levels and affect RES-induced catalepsy on rats. Our study involves exploring the cellular processes via which Afzelin works to unveil possible therapeutic implications in tackling catalepsy. This study will also contribute to the exploration of more information about catalepsy, giving us better insights into this complicated phenomenon. We are therefore able to understand how cellular mechanisms causing catalepsy can also be affected with afzelin and thus provide some therapeutic value against this condition. This will lead to generation of new evidence that could help develop new therapy interventions and further research on Afzelin as a drug for treatment of catalepsy.

## 2 Material and methods

### 2.1 Drugs

This study involved the induction of a catalepsy by injecting them 1 mg/kg ip dose of RES (St. Louis, Missouri, U.S.A.). The RES solution was made by diluting it using 0.5 % glacial acetic acid and distilled water prior to its administration. Levodopa from Sun Pharmaceutical Industries Ltd., Mumbai, India was used for comparison. The levodopa dosage delivered to each rat was 30 mg/kg ip. Afzelin was an experimental drug bought from Sigma, located in St. Louis, MO, USA. For this study, rats were given oral doses of Afzelin 5, 10 and 20 mg/kg. High-quality analysis Chemicals and solvents were used to achieve precision and reliability in the results. The detection of Bcl-2 proteins was performed using the Thermo Scientific ELISA kit in Rockford, IL, USA. All the necessary tools for accurate determination of protein amounts were supplied with the instructions for use in these kits.

### 2.2 Animals

The experimental animals for this study were female Sprague-Dawley rats that weighed between 190 and 220 g. The approval for conducting ethical issues in this study was granted by SCBR-037-2022, which is from the Standing Committee on Bioethical Research at the College of Pharmacy, Prince Sattam Bin Abdul Aziz University in Saudi Arabia. Next, the rats were kept in a rodent quarantine center for one week and transferred to the animal house. A standard 12-hour diurnal cycle was used in the exposure of rats.

Different groups were randomly allocated to rats:

Group I: Control group, received only the vehicle (no drugs administered).
Group II: RES was injected intraperitoneally (1 mg/kg) into this control group for alternative 5 days, day 3, 5, 7, 9 and 11.
Group III: Standard therapy received levodopa 30 mg/kg and RES 1 mg/kg i.p.
Group IV: 5 mg/kg of Afzelin orally, daily and RES as in control group.
Group V: Afzelin 10 mg/kg orally, daily and RES as in control group.
Group VI: Afzelin 20 mg/kg orally, daily and RES as in control group.

RES-induced catalepsy was examined in rats in this research. On alternate days for 3, 5, 7, 9, and 11 days, 1 mg/kg RES was injected intraperitoneally. The rats' cataleptic behaviour and aberrant movements were induced by this therapy. On days 14 and 15, the rats were tested in various behavioural studies. These tests included the open-field test, which measures exploratory behaviour and locomotor activity, the modified forced swim test, which measures despair-like behaviour, and the rota rod test, which measures motor coordination and balance. These tests looked for behavioural signs of catalepsy. Behavioral testing was conducted on the rats, which led to their euthanasia and careful extraction of their striatum and hippocampus. The quality of tissue samples for future chemical analyses of the brain was maintained by freezing them at −70 °C. Therefore, this experiment aimed to examine the effects of any such change in the chemical composition of the striatum and hippocampus on cataleptic symptoms induced by RES in our rat model.

#### 2.2.1 Open field test

One of the standard tools used for behavioural research to assess movement or behavioural activity as well as animals’ reactions to stress is the open field test (OFT). The current study is essential in informing us how animals behave under varying treatment modalities. We used OFT on days seven and fourteen to monitor any changes in motor behavior. The test arena comprised of a round enclosure of 97 cm in diameter by 42 cm high. It had three peripheral zones that were marked out, and nineteen sub quadrants for accurate study of animal movements. After each trial, the open filed apparatus was well washed with a 5 % water and alcohol mixture so as to eliminate all possible animal or other body smells in order to guarantee accurate results. Prior to the actual test, the animals were put into the central portion of the testing arena, provided with five minutes to freely wander about, and then allowed to settle down. Observations were observed and recorded carefully in line with measurement of animal’s movement, rearing as well as immobility during the whole experiment. The recording were conducted in order, to have a wholesome perception of the movement undertaken by the animals, when tested (Võikar and Stanford, 2023).

### 2.3 Modified forced swim test

Modified compelled swim test (MFST) is an essential, which helps in assessing therapeutic effects of various interventions and drugs towards behavioral examinations. For this experiment, rats were used as research subjects whereby they went through the MFST process on day seven and fourteen. The rats were housed in a tank maintained at 24 ± 1℃, containing water at 30 cm depth (for 15 min/day). On the following day, the subjects underwent five-minute trials. Distinct behavioural parameters of the rats were carefully observed and measured during the experimental session. Three critical parameters were assessed: walking or immobility, swimming, and climbing behaviour. Immobility was distinguished as a total lack of physical activity. Swimming was more significant, pushing water over the minimum level necessary to support head floating. Climbing behavior movements occurred both inside and out of the water as well, targeting the walls. Replacement of the water in the tank was done on a rat basis to eliminate any possible bias. This protocol ensured that long-term effects from other rats did not interfere with the behaviour of other experimental subjects (Tanaka et al., 2020, Woźniak et al., 2018, Yankelevitch-Yahav et al., 2015).

### 2.4 Motor co-ordination test (Rota-Rod test)

Rotarod test is an accepted scientific technique used in evaluating motor coordination and balance in rodents. The experimental procedure involves putting rats in a rotating cylindrical drum whose speed is increased gradually. However, in this experiment, motor coordination was evaluated using a Rota-Rod model on Days eight and fifteen. During this experiment, the rats were placed upon the Rota-Rod’s rotating arm for one minute. The purpose of the study was to see how long the rats could maintain their balance on a rotating cylinder for 60 s (one minute). Motor coordination was quantified in terms of the rat’s loss of balance, falling from the wheel once it had stopped turning (Carter et al., 2001, Deacon, 2013, Luong et al., 2011).

### 2.5 Biochemical analysis

#### 2.5.1 Estimation of antioxidant level in tissue homogenates

It uses spectrometry to evaluate the extent in which superoxide dismutase (SOD) hinders adrenaline oxidation into adrenochrome. SOD is able to interact with superoxide radicals and produce H_2_O_2_ and O_2_ molecules. Distinguishable from Hydrogen Peroxide is Adrenochrome which adsorbs the light at the wavelength of 480 nm. The high levels of SOD consequently lead to lower levels of adrenochrome. The experiment used 0.05 mL supernatant, 2 mL of carbonate buffer and 0.5 mL of EDTA. Such findings offer an insight on SOD’S action on some of the oxidative reactions in this biochemical way. At pH 10.2, epinephrine (0.5 mL) oxidized adrenaline (3 × 10^−4^ M) to form adrenochrome. The optical density changes at 480 nm, indicating adrenochrome production, were monitored every minute and compared against a reagent blank. The CAT activity assay followed the instructions provided in the kit. Absorbance at 240 nm was recorded every 10 s for 1 min. The results were expressed as activity units per milligram of protein. For GSH determination, kits were used and the manufacturer's manual was followed. The absorbance was measured and the results were expressed as micromoles per gm of tissue.

#### 2.5.2 Estimation of lipid peroxidative assay

The Niehaus-Samalesson lipid peroxidation method was used to measure it. This procedure is used to quantify the chemicals that react with thiobarbituric acid. In particular, 0.1 mL of homogenate sample was combined with thiobarbituric acid, trichloroacetic acid, and hydrochloric acid in precise quantities. The reagent solution included 0.37 % TBA, 15 % TCA and 0.25 N HCl. Following the addition of the reagent to the sample, it underwent an incubation period lasting for 15 min within a constant temperature water bath. Subsequently, cooling measures were implemented followed by centrifugation under room temperature conditions using gravitational force equivalent to that achieved under spinning at approximately one-thousand times Earth's gravity or around1000 g-forces for ten minutes. The resulting clear supernatant obtained after centrifugation was examined by measuring its absorbance reading against a reference blank utilising spectrophotometric techniques specifically set to wavelengths equal to 535 nm.

#### 2.5.3 Determination of nitric oxide (NO)

Specifically, in this research study assessments of nitric oxide (NO) levels were undertaken using the acidic Griess reaction analytical method. Nitrate was reduced using vandium trichloride which converted it into nitrite for more accurate measurements. A colourimetric reaction that occurs when the mixture of nitrite, sulfonamide, and N-(1-naphthyl) ethylenediamine is referred to as the Griess reaction. A pink azo, which absorbs light at a particular wavelength of 543 nm, results from this chemical reaction. Reportable accuracy was obtained by comparing the nitrate concentrations of samples against a standardized sodium nitrates curve and expressed in micrograms per milliliter (µg/ml) units (Yao et al., 2019).

#### 2.5.4 Estimation of the biogenic amine

This parameter was carried out as per the procedure published by Biradar et al. (2012). In the current investigation, animals were sacrificed as per the approved protocol and brain tissue was homogenized in a hydrochloric acid–butanol solution. After centrifugation, the supernatant was mixed with n-heptane and hydrochloric acid. Following separation, the aqueous phase received EDTA/Sodium acetate buffer and iodine for oxidation. The reaction was stopped, and acetic acid was added before heating the solution. Excitation and emission spectra were recorded for Noradrenaline and Dopamine. Values indicated the concentration of biogenic amines, and the brain's amine content was calculated (Biradar et al., 2012).

#### 2.5.5 Estimation of Bcl-2 with rat ELISA kit

The tissue homogenates were sonicated to disrupt cells. After sonication, the homogenates were centrifuged at 5000 × g for 5 min. The liquid supernatant was carefully taken. The documentation instructed me to freshly produce the buffer, standard working solution, biotinylated detection antibody working solution, and HRP conjugate working solution. In each well, 100 μL of either the standard or sample was dispensed. Subsequently, the plate was placed in an incubator at 37° C for 90 min. After the specified incubation period, the liquid was aspirated and 100 μL of biotinylated detection antibody was introduced. An additional incubation of one hour at 37° C was carried out. Subsequently, the wells were aspirated and washed three times with wash buffer. Subsequently, 100 μL of HRP conjugate was added to each well, and the plate was subjected to a 30 min incubation at 37° C. After aspirating once more and conducting five additional washes, 90 μL of substrate reagent was introduced into each well. The plate was then incubated at 37° C for 15 min. Subsequently, 50 μL of Stop Solution was added and the absorbance of the plate was immediately measured at 450 nm.

### 2.6 Statistics

The data collected from the open field test were analyzed using a two-way analysis of variance (ANOVA) in the past tense. In this analysis, the group was considered as the between-subjects factor, and time was treated as the within-subjects factor. Following this analysis, multiple comparisons were carried out utilizing the Newman-Keuls test.

## 3 Results

### 3.1 Open field test

**Fig. 1:**
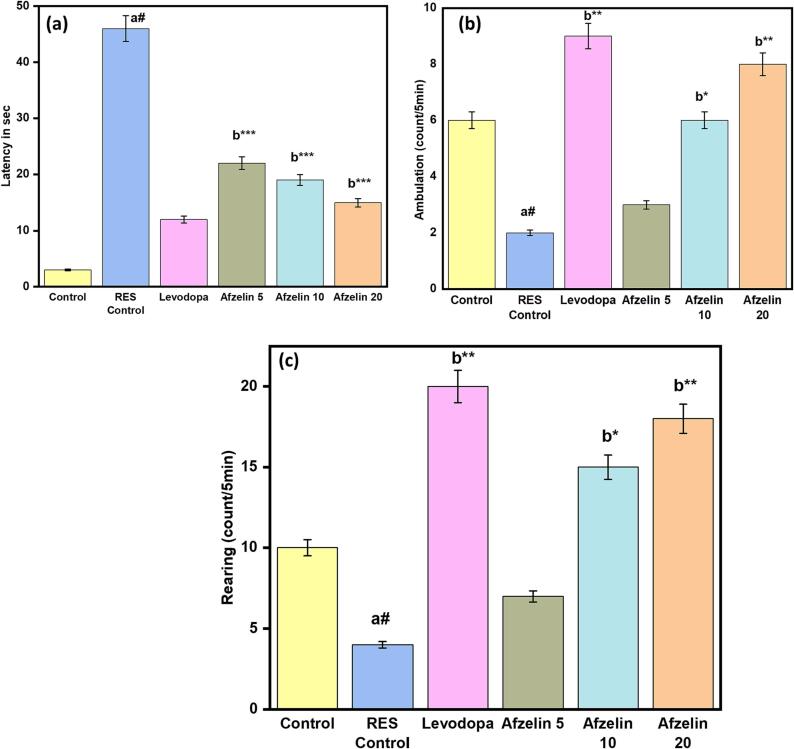
Effect of Afzelin on behavioral changes in open field test. (a) Latency period; (b) Ambulation and (c) Rearing time. The statistical significance threshold was set at a p-value of less than 0.05. Comparison between normal control vs RES control denoted with (a), # p < 0.001; and RES control vs treatment groups were denoted with (b), ***p < 0.001, ** p < 0.01, *p < 0.05 analyzed for significant differences.

The open field test revealed interesting findings across different treatment groups as shown in Fig. 1. The control group exhibited quick entry into the field (3 s), high ambulation count (7), and substantial rearing behavior (10), indicating increased locomotor activity and exploration. In contrast, the RES control group showed delayed entry (46 s), reduced ambulation (2), and rearing (4), indicating decreased locomotor activity and exploration. Levodopa administration increased locomotor activity (latency: 12 s, ambulation: 9) and exploratory behavior (rearing: 20). Afzelin treatment showed a dose-dependent effect, with lower doses decreasing latency (Afzelin 5: 28 s) and higher doses promoting both ambulation (Afzelin 10: 8, Afzelin 20: 10) and rearing behavior (Afzelin 10: 14, Afzelin 20: 17). These results provide valuable insights into the effects of different treatments on behavior in the open field test paradigm.

### 3.2 Modified forced swim test

**Fig. 2:**
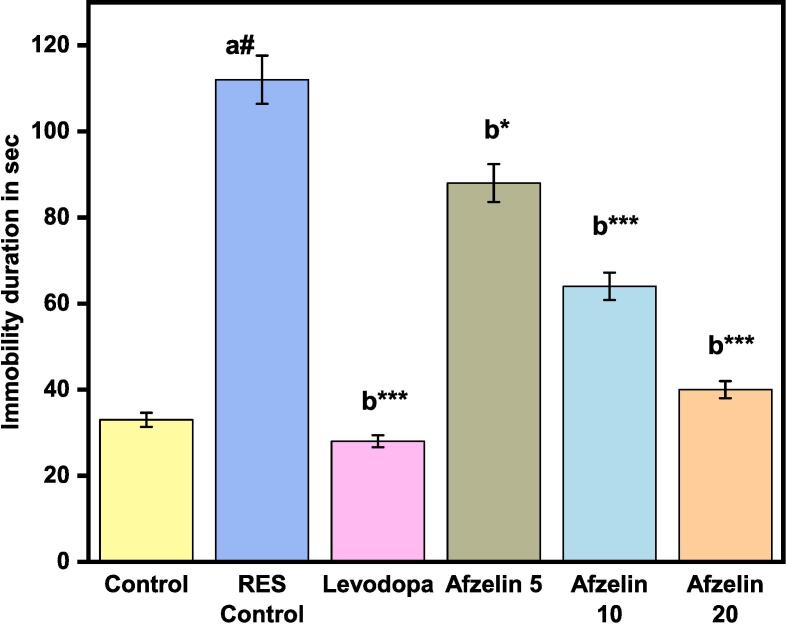
Effect of Afzelin on Immobility in Modified Forced Swim test. The statistical significance threshold was set at a p-value of less than 0.05. Comparison between normal control vs RES control denoted with (a), # p < 0.001; and RES control vs treatment groups were denoted with (b), ***p < 0.001, *p < 0.05 analyzed for significant differences.

Fig. 2 shows the effects of Afzelin (5, 10 and 20 mg/kg, p.o.) and levodopa (30 mg/kg, p.o.) on the duration of immobility in the forced swimming test. To evaluate an experimental condition or therapy, immobility is evaluated in seconds. Drugs and control groups were tested for immobility duration. In the control group, baseline immobility was 33 s. The RES control group, which got a different treatment or intervention, had 112 s of immobility, showing a greater amount of immobility than the standard control. Levodopa, a Parkinson's drug, reduced immobility to 28 s. Levodopa may improve movement and reduce immobility. The research examined the effects of varying Afzelin concentrations. Afzelin 5, 10, and 20 mg had immobility durations of 86.4 s, 64.2 s, and 38.5 s, respectively. These data imply that raising Afzelin concentration reduces immobility time, suggesting a dose-dependent mobility effect. These consecutive data show how treatments and drugs affect immobility length. Levodopa and greater Afzelin concentrations enhanced mobility.

### 3.3 Rota rod test

**Fig. 3:**
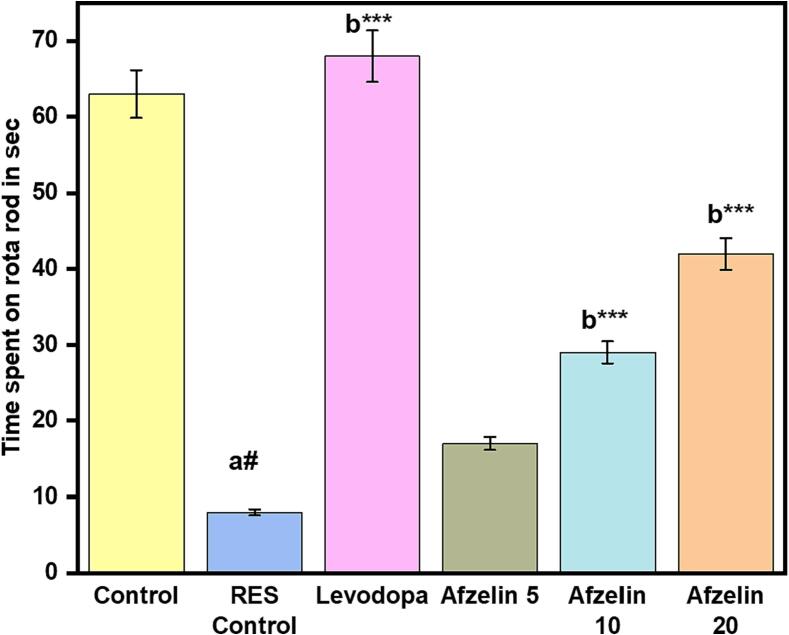
Effect of Afzelin on time spent on the Rota rod. The statistical significance threshold was set at a p-value of less than 0.05. Comparison between normal control vs RES control denoted with (a), # p < 0.001; and RES control vs treatment groups were denoted with (b), ***p < 0.001 analyzed for significant differences.

The Rota rod test is used to evaluate animal motor coordination and balance in neurological studies. This research used the Rota rod test to measure the animals' time on the spinning rod in seconds. The baseline performance, the control group, averaged 63 s on the rota rod. This shows that the control group's animals-maintained balance and coordination for a long period. The RES control group, exhibited a significant loss in motor coordination. These animals spent an average of 8 s on the rota rod, demonstrating poor balance and coordination compared to the control group. Levodopa, a Parkinson's disease drug, was administered to another group to reduce motor symptoms. Levodopa improved motor coordination and balance in this group's rota rod time of 68 s. Motor performance was also tested with various doses of Afzelin (5, 10, and 20 mg). Compared to the RES Control group, the Afzelin 5 animals spent 17.5 s on the rota rod, demonstrating improved motor coordination as shown in Fig. 3. Animal performance enhanced with Afzelin dosage. The Afzelin 10 group spent 29.8 s on the rota rod, whereas the Afzelin 20 group spent 43.7 s, showing a dose-dependent impact on motor coordination. These results demonstrate the relevance of the Rota rod test in assessing motor coordination and balance in experimental animals. The RES control group and the animals receiving lesser doses of Afzelin had poor motor function, whereas the control group performed well.

### 3.4 Antioxidant analysis

**Fig. 4:**
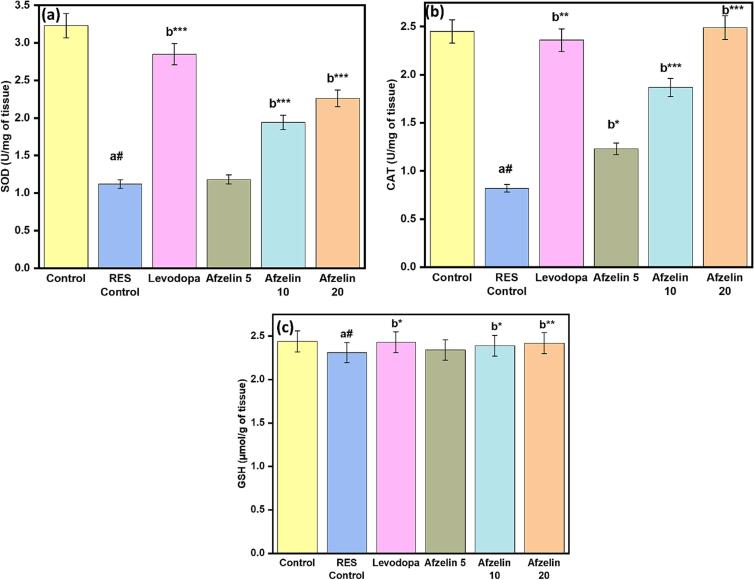
Effect of Afzelin on antioxidant parameters. (a) SOD, (b) CAT and (c) GSH. The statistical significance threshold was set at a p-value of less than 0.05. Comparison between normal control vs RES control denoted with (a), # p < 0.001; and RES control vs treatment groups were denoted with (b), ***p < 0.001, ** p < 0.01, *p < 0.05 analyzed for significant differences.

Antioxidant analysis measured SOD, CAT, and GSH in tissue samples. The control group had the highest SOD activity (3.23 U/mg tissue), suggesting strong protection against superoxide radicals. However, the RES control group exhibited reduced SOD activity (1.12 U/mg of tissue), which could affect antioxidant action. Levodopa showed moderate antioxidant effects, with SOD activity at 2.85 U/mg of tissue as shown in Fig. 4. Afzelin at higher doses (10 and 20 mg) positively influenced SOD activity, leading to enhanced defence against superoxide radicals. CAT activity in the control group was 2.45 U/mg of tissue, efficiently breaking down hydrogen peroxide. However, the RES control group showed decreased CAT activity (0.82 U/mg tissue), potentially affecting hydrogen peroxide detoxification. Levodopa resulted in a slight increase in CAT activity (2.66 U/mg of tissue), suggesting a positive effect on hydrogen peroxide detoxification as shown in Fig. 4. Similar increases in CAT activity were observed at higher doses of Afzelin (10 and 20 mg). Differences in GSH levels were small between groups, indicating a lack of therapeutic efficacy. On the other hand, Levodopa and larger doses of Afzelin showed promise in improving antioxidant defence. The investigation sheds information on the potential of Levodopa and higher doses of Afzelin by revealing their impacts on antioxidant enzyme levels. More studies are needed to determine if and how they might be used in treatment.

### 3.5 TBARS in the brain of RES-treated rats

**Fig. 5:**
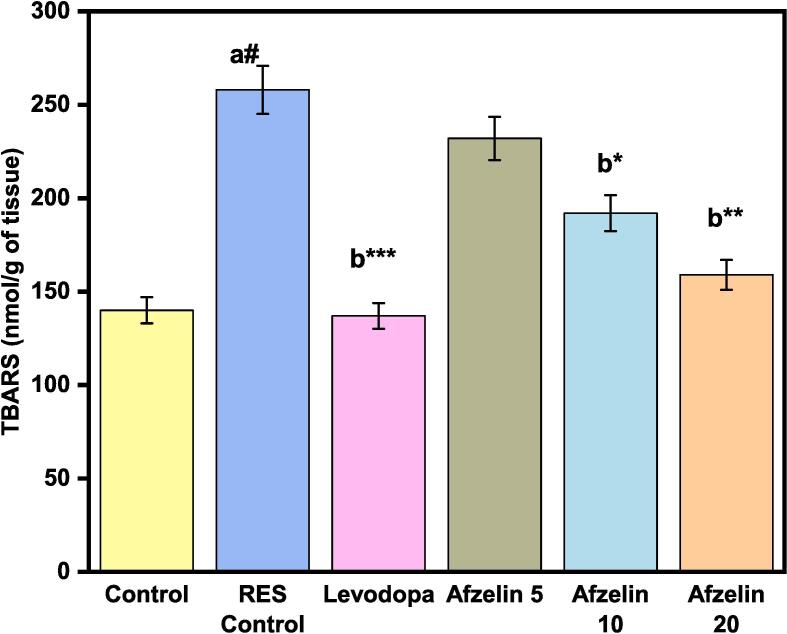
Effect of Afzelin on TBARS. The statistical significance threshold was set at a p-value of less than 0.05. Comparison between normal control vs RES control denoted with (a), # p < 0.001; and RES control vs treatment groups were denoted with (b), ***p < 0.001, ** p < 0.01, *p < 0.05 analyzed for significant differences.

The study measured TBARS levels in the brains of rats treated with RES, which serve as a marker of oxidative stress. The control group showed relatively low oxidative stress with a TBARS level of 140 nmol/g of tissue. On the contrary, the RES control group treated with RES showed significantly higher oxidative stress with a TBARS level of 258 nmol/g of tissue, indicating increased lipid peroxidation. Treatment with levodopa did not significantly affect oxidative stress, as evidenced by a TBARS level of 137 nmol/g of tissue, similar to the control group. However, different doses of Afzelin revealed varying effects. Afzelin at 5 mg led to increased oxidative stress (TBARS level of 212 nmol/g tissue), while higher doses of Afzelin (10 and 20 mg) potentially provided protection, showing lower TBARS levels of 194 and 148 nmol/g tissue, respectively, as shown in Fig. 5. In conclusion, RES treatment increased oxidative stress in the brain, Levodopa had no notable effect, and higher doses of Afzelin may offer protection against oxidative damage. These results shed light on the impact of treatments on oxidative stress and highlight the potential of afzelin as a protective agent in mitigating oxidative damage in the brain.

### 3.6 Nitric oxide level in brain homogenates

**Fig. 6:**
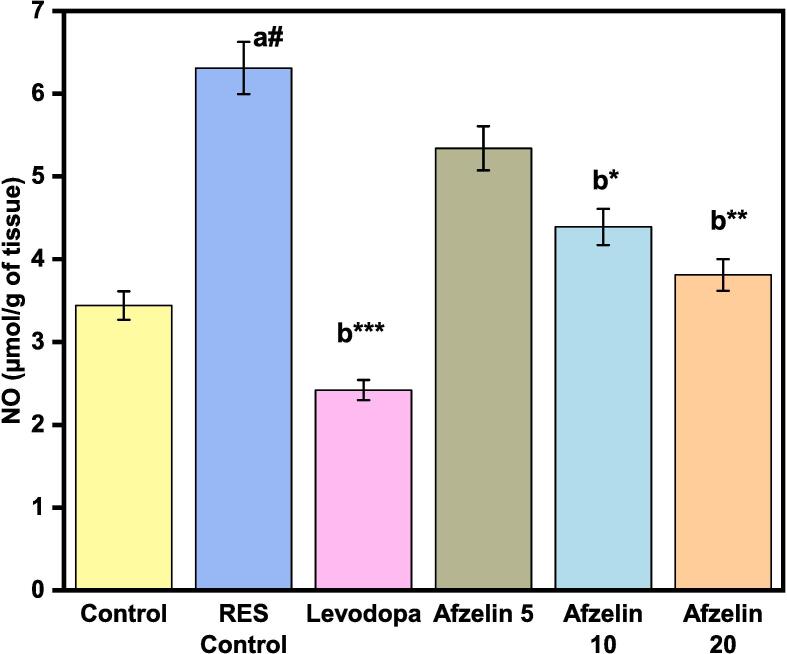
Effect of Afzelin on Nitric oxide level in tissue homogenates. The statistical significance threshold was set at a p-value of less than 0.05. Comparison between normal control vs RES control denoted with (a), # p < 0.001; and RES control vs treatment groups were denoted with (b), ***p < 0.001, ** p < 0.01, *p < 0.05 analyzed for significant differences.

The research found substantial differences in brain homogenate nitric oxide (NO) levels between the treatment groups. NO was 3.44 μmol/g in the control group. RES treatment led to a notable increase in NO levels (6.31 µmol/g tissue), while Levodopa showed a mild suppressive effect (2.42 µmol/g tissue). Furthermore, the groups treated with different doses of Afzelin demonstrated distinct responses. Afzelin at 5 mg resulted in elevated NO levels (5.34 µmol/g tissue), while higher doses (10 and 20 mg) led to reduced NO levels (4.39 and 3.81 µmol/g tissue, respectively) as shown in Fig. 6. These results provide information on how brain nitric oxide synthesis is affected by therapies. RES increased NO levels, Levodopa slightly decreased them, and Afzelin had dose-dependent effects.

### 3.7 Effect of Afzelin on RES-induced alterations in brain monoamines

In rats treated with RES, there was a significant reduction (p < 0.05) in levels of dopamine (DA), norepinephrine (NA), and serotonin (5-HT) compared to normal rats. Administration of Afzelin at doses of 5, 10, and 20 mg/kg significantly alleviated (P < 0.05) the decrease in these RES-induced biogenic monoamines in brain homogenates. Treatment with levodopa at a dose of 30 mg/kg also significantly increased (p < 0.05) the levels of DA, NA and 5-HT levels compared to the control rats of RES represented in Table 1. However, the decrease in DA, NA and 5-HT levels induced by RES was significantly more inhibited (p < 0.05) with Afzelin treatment at 20 mg/kg compared to treatment with levodopa at 30 mg/kg.

**Table 1:** Effect of Afzelin on RES-induced alterations in brain monoamines.

	Dopamine (µg/g of tissue) (mean ± SEM)	Serotonin (µg/g of tissue) (mean ± SEM)	Noradrenaline (µg /g of tissue) (mean ± SEM)
Control	0.45 ± 0.08	0.24 ± 0.02	0.14 ± 0.03
RES Control	0.34 ± 0.07	0.13 ± 0.09	0.03 ± 0.01
Levodopa	0.42 ± 0.06	0.35 ± 0.03	0.11 ± 0.02
Afzelin 5	0.35 ± 0.03	0.23 ± 0.11	0.07 ± 0.03
Afzelin 10	0.39 ± 0.05	0.36 ± 0.06	0.10 ± 0.02
Afzelin 20	0.41 ± 0.03	0.39 ± 0.05	0.12 ± 0.03

### 3.8 Effects of Afzelin on Bcl-2

**Fig. 7:**
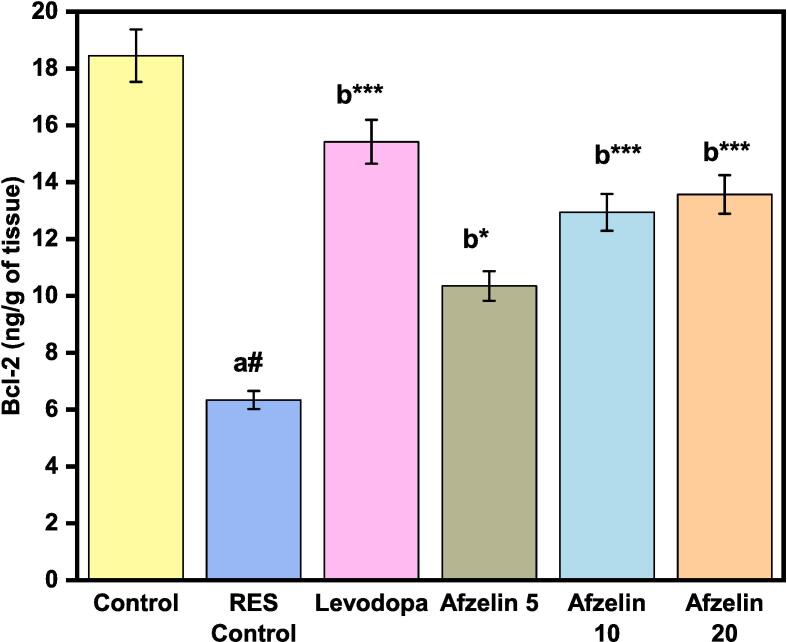
Effect of Afzelin on Bcl-2 level in tissue homogenates. The statistical significance threshold was set at a p-value of less than 0.05. Comparison between normal control vs RES control denoted with (a), # p < 0.001; and RES control vs treatment groups were denoted with (b), ***p < 0.001, *p < 0.05 analyzed for significant differences.

The Bcl-2, a protein that controls apoptosis and estimated in tissue homogenates. RES treatment reduced Bcl-2 levels to 6.34 ng/g tissue from 18.45 ng/g tissue in the control group. Afzelin inhibits Bcl-2, perhaps affecting apoptotic signalling pathways. Levodopa treatment raised Bcl-2 levels to 15.42 ng/g tissue, somewhat lower than the control group but higher than the RES Control group. Afzelin doses affected Bcl-2 expression differently. Afzelin 5 decreased Bcl-2 levels (10.35 ng/g tissue) compared to the control group, but Afzelin 10 and 20 raised Bcl-2 (12.47 and 14.10 ng/g tissue) as depicted in Fig. 7. These data suggest that higher Afzelin doses may upregulate Bcl-2.

## 4 Discussion

According to current research findings, the administration of Afzelin was found to be effective in ameliorating motor and kinesia deficits caused by RES. This animal model is commonly used to simulate both PD and dyskinesia. The research utilized the OFT, FST, and Rotarod test as the primary behavioral assessment tools. The measurement of locomotor activity was employed as a means to assess cognitive vigilance, as numerous substances that impact the central nervous system (CNS) also have an effect on locomotor activity. The primary aim of this investigation was to evaluate the influence of Afzelin on the locomotor activity of rats by employing the OFT. The findings demonstrated significant stimulatory effects on the central nervous system of Afzelin, as evidenced by the observed increase in locomotor activity in rats. Increased locomotor function is a reliable indicator of heightened alertness, whereas a reduction in locomotor function is suggestive of sedative activity. The FST and TST are established models used for assessing the antidepressant effects. These tests replicate despair behaviors in animals and simulate a state of depression. The immobility state induced by FST and TST in animals closely mimics human depression and can be reversed with antidepressant medications. The recent findings offer compelling evidence of Afzelin's significant anti-cataleptic effect.

RES did not show any influence on anxiety-related behavior or learning, but it did negatively affect memory performance in the discriminative avoidance task. These findings align with prior studies conducted on rats and mice (Peres et al., 2016, Silva et al., 2002). Significantly, the noticeable influence of RES on locomotor activity and movements was observed only on day 5, following the second injection of the substance. Similarly, the statistically significant impact on catalepsy occurred only on day 7, 48 h after the second injection. Additionally, the observable effect on memory became apparent 96 h after the second injection. The gradual onset of motor abnormalities and memory impairments indicates a slow and non-acute progression of RES's influence. Consequently, this effect is more likely associated with oxidative damage rather than the acute depletion of monoamines caused by RES.

At the neurochemical level, the treatment of RES led to a notable decrease in the concentrations of monoamine neurotransmitters (namely, norepinephrine, serotonin, and dopamine) in both the cortex and hippocampal regions. Several studies have provided empirical evidence supporting the idea that the disruption of cerebral monoaminergic activities is a fundamental mechanism contributing to the onset of depression and catalepsy. In rats, the effects of RES include various manifestations such as hypothermia, akinesia, and ptosis (El-Marasy et al., 2021). In the experiment investigating the impact of RES on hypothermia antagonism and ptosis behavior, Afzelin displayed significant inhibitory effects on both hypothermia and ptosis in RES-induced rats. The results of this study align with the known clinical effectiveness of Afzelin in the treatment of depression. The findings suggest that Afzelin's anticateleptic-like effect might be linked to the catecholamine and/or serotonin systems. Other studies have also shown that quercetin, (-) epigallocatechin 3-gallate, and cannabidiol can improve memory and learning in animals treated with RES and have a reduced AChE activity, decreased oxidative stress, and mitigated (Naidu et al., 2004, Peres et al., 2016, Samad et al., 2021, Tseng et al., 2015). Afzelin from *Ribes fasciculatum* was studied for its neuroprotective potential against cognitive dysfunctions and memory deficits in mice. The cognitive and memory functions are affected by the neurodegenerative diseases through the low BDNF and CREB signaling. To elicit cognitive impairment in mice, scopolamine and afzelin were administered via injection into their third ventricles. The findings revealed that the introduction of afzelin in scopolamine-treated mice resulted in heightened synaptic plasticity and improved cognitive function. Afzelin exhibited favorable effects on the cholinergic systems and CREB-BDNF signaling pathways, thereby contributing to augmented neurocognition and neuroprotection (Oh et al., 2021). However, it is important to note that Afzelin demonstrates robust antioxidative properties, thereby safeguarding cognitive function that might otherwise be compromised by the detrimental impact of RES.

Recent research has elucidated that Reactive Oxygen Species (ROS) are also implicated in the pathogenesis of depressive or dyskinesia disorders (Madireddy and Madireddy, 2020, Murray et al., 2021). Moreover, initial investigations have suggested that the modulation of oxidative stress could play a role in the therapeutic efficacy of certain antidepressant medications (Correia et al., 2023). However, the exact mechanism by which Afzelin exerts its anticateleptic property is not fully explained. To enhance Afzelin's anticateleptic processes, a study was undertaken to examine its impact on oxidative stress in the striatum of rats subjected to RES treatment. The findings of the study demonstrated that the administration of Afzelin successfully decreased the heightened level of TBARS caused by RES in tissue homogenates (p < 0.01). In contrast, no statistically significant distinction was observed between the efficacy of levodopa therapy and Afzelin. This observation indicates that the anticateleptic mechanism of Afzelin bears resemblance to that of Levodopa. Cells possess an inherent antioxidant defense mechanism that serves to eliminate ROS in order to safeguard against potential harm to cellular structures. The cellular antioxidant defenses typically function to regulate the quantities of ROS within cells, thereby preventing excessive oxidation of biological molecules. Endogenous antioxidants like SOD, GSH, CAT, etc., protect cells from damage. The antioxidant defense system relies heavily on enzymes like SOD. The superoxide radical is neutralized as it is transformed into oxygen and hydrogen peroxide. In the presence of GSH or CAT, H_2_O_2_ can be degraded to water. GSH is a key biomolecule in the body's antioxidant defense mechanism. The afzelin in plant extracts functions as a powerful antioxidant and lipid peroxidation inhibitor. Furthermore, Afzelin significantly restored the significantly lowered levels of endogenous antioxidant enzymes (SOD, GSH-Px, and CAT) in the depressed animals produced by RES. These findings are consistent with a prior investigation that found Afzelin to be effective in increasing antioxidant capabilities in the face of oxidative stress. These findings suggest that Afzelin can counteract the oxidative stress caused by catalepsy in the rat striatum via regulating the levels of ROS and antioxidant enzymes.

ROS are essential cellular signals, but an imbalance can cause diseases. NOX enzymes solely produce ROS, with both beneficial and harmful effects. Elevated NOX activity contributes to diseases like cardiovascular issues, neurodegeneration, and cancer. Natural compounds, especially polyphenols, can target NOX without disrupting the body's redox balance. This review highlights NOX's roles in physiology and pathology and explores natural NADPH oxidase inhibitors, like polyphenols (Björkman et al., 2018, Maraldi, 2013, Yousefian et al., 2019, Foudah et al., 2022). We have found out that Afzelin can regulate NO production and function which is one of the important signal messengers taking part in many physiologic effects. It is important to maintain proper control for dysregulation of NO homeostasis could induce oxidative stress and cellular damage.

During our investigation, we established that administration of Afzelin increased the level of expression of the protective protein Bcl-2 in the striatum. Bcl-2 is involved in regulating neuron death. This is the first documented mechanism describing how Afzelin spares the striatal neurons from apoptosis caused by oxidative stress, in rats. This research shows how important afzelin is for protecting a person from cataleptic conditions, mostly considering it as antioxidant effect.

The exact process through which RES causes cataplexy is still vague. However, new research has shed light on the possibility of such a role played by Bcl-2 – a well-known anti- apoptotic protein renowned for its paramount function in survival of cells and safety of brain against injury. Another possible explanation is that higher levels of expression of the protein Bcl-2 could have quite an effect on this phenomenon. In essence, Bcl-2 prevents cytochrome *c* release out of mitochondrion that stops a certain type of cell passing programmed death called apoptosis. It may appear surprising given the extensive studies conducted on the subject of cancer or neurodegenerative disorder before this finding. Using the unique ability of the Afzelin to influence the oxidative stress pathways that involve the oxidative radicals implicated in PD, it is possible to explain the neuroprotective mechanisms of the plant. Unlike most other drugs Afzelin becomes important in neuroinflammation inhibition and mitochondria enhancement for treatment of different elements of pathophysiology (Tan et al., 2022). Moreover, Afzelin exhibits a notable capacity to traverse the blood–brain barrier with efficiency, thereby enhancing its potential as an efficacious and precisely targeted therapeutic agent in the context of PD.

Further, the research implies that Afzelin has potential therapy for PD. Afzelin modulates levels of neurotransmitters, regulates antioxidant defense mechanisms, and promotes anti-apoptotic pathway via Bcl-2. Further research in this area may focus on the different molecular pathways involved, assessing of the same or better effectiveness than other established treatments, and possible use in other indications in addition to obesity. However, its role in the development of therapeutic intervention against PD also needs to be demonstrated by explaining the precise mechanisms through which it works and how exactly its action is directed towards the mitochondria.

## 5 Conclusion

This study investigates Afzelin's neuroprotective properties in a rat model of RES-induced catalepsy, which resembles PD. Current PD medicines manage symptoms rather than preventing disease development. The research investigated Bcl-2, an anti-apoptotic protein, and Afzelin's neuroprotective and striatal oxidative stress-reducing effects. The outcomes of the study revealed that Afzelin treatment enhanced Bcl-2 expression in the striatum, a brain region affected in PD. This upregulation of Bcl-2 may contribute to Afzelin's protective effects against cataleptic symptoms induced by RES. Furthermore, Afzelin has been shown to exhibit antioxidative properties, exerting modulation over ROS production and levels of antioxidant enzymes in the striatum. This observation implies its potential role in mitigating oxidative stress. The deductions drawn from this research underscore the conceivable therapeutic advantages of Afzelin in addressing neurodegenerative disorders, such as PD. Its ability to enhance Bcl-2 expression and reduce oxidative stress positions Afzelin as a potential neuroprotective agent. Future research into the exact mechanism through which Afzelin produces these symptoms should be directed towards the generation of new anti-cataleptic medications for neurodegenerative disorders. However, further comprehensive investigations should be made to clearly define the mechanism(s) of action through which Afzelin can be utilized clinically to manage PD-related degeneration. Therefore, this study highlights the potential of focusing on natural compounds including Afzelin as possible neuroprotective agents. This approach provides various ways for enhancing effectiveness of treatment of PD.

## Ethical approval

Ethical approval for conducting this research was granted by the Standing Committee on Bioethical Research (SCBR-037-2022) at the College of Pharmacy, Prince Sattam bin Abdulaziz University in Saudi Arabia.

## Acknowledgments

This study was supported via funding from Prince Sattam bin Abdulaziz University Project Number PSAU/2023/R/1445.

## Declaration of competing interest

The authors declare that they have no known competing financial interests or personal relationships that could have appeared to influence the work reported in this paper.
